# Duration and Quality of Sleep and Risk of Physical Function Impairment and Disability in Older Adults: Results from the ENRICA and ELSA Cohorts

**DOI:** 10.14336/AD.2018.0611

**Published:** 2019-06-01

**Authors:** Marcela Z. Campanini, Arthur E. Mesas, Jose Antonio Carnicero-Carreño, Fernando Rodríguez-Artalejo, Esther Lopez-Garcia

**Affiliations:** ^1^Department of Public Health, Universidade Estadual de Londrina, Londrina, Brazil.; ^2^Department of Preventive Medicine and Public Health, School of Medicine, Universidad Autónoma de Madrid/IdiPaz, Spain.; ^3^Foundation for Biomedical Research, Getafe University Hospital, Getafe, Spain.; ^4^CIBER of Epidemiology and Public Health, Madrid, Spain.; ^5^IMDEA-Food Institute. CEI UAM+CSIC, Madrid, Spain.

**Keywords:** physical function, sleep, physical activity.

## Abstract

Sleep duration and quality have been associated with poor physical function, but both the temporality of the association and the independence of sleep duration and quality are unclear. We examined the prospective association of sleep duration and quality with physical function impairment and disability in older adults. Data were taken from participants in the Seniors-ENRICA (2012-2015, n= 1,773) and in the ELSA cohort (waves 4 and 6, n=4,885) aged ≥60 years. Sleep duration and quality were self-reported. Physical function impairment and disability was obtained either from self-reports (ENRICA and ELSA) or from performance assessment (ENRICA). Logistic regression models were adjusted for potential confounders. After a follow-up of 2.0-2.8 years, no association was found between changes in sleep duration and physical function impairment or disability. However, in both studies, poor general sleep quality was linked to higher risk of impaired agility [OR: 1.93 (95% CI: 1.30-2.86) in Seniors-ENRICA and 1.65 (1.24-2.18) in ELSA study] and mobility [1.46 (0.98-2.17) in Seniors-ENRICA and 1.59 (1.18-2.15) in ELSA study]. Poor general sleep quality was also associated with decreased physical component summary (PCS) [1.39 (1.05-1.83)], disability in instrumental activities of daily living [1.59 (0.97-2.59)] and in basic activities of daily living [1.73 (1.14-2.64)] in Seniors-ENRICA. In addition, compared to those with no sleep complaints, participants with 2 or more sleep complaints had greater risk of impaired agility, impaired mobility, decreased PCS and impaired lower extremity function in both cohorts. Poor sleep quality was associated with higher risk of physical impairment and disability in older adults from Spain and from England.

Limitation in physical functioning and disability are important public health problems because of their increasing frequency, linked to population aging, and because they are associated with greater risk of morbidity, hospitalization and death (1,2) as well as elevated healthcare expenditures (3).

Sleep duration and sleep quality are two of the potentially modifiable factors that have been associated with physical function impairment, disability and related factors. Specifically, short sleep duration has been associated with bodily pain (4), mobility limitation (5) and disability (6), and long sleep duration with decreased gait speed (5) and physical function decline (7) in older adults. In addition, poor sleep quality has been related to lower body functional capacity (5, 8, 9), frailty (10,11) and disability (12).

A better understanding of the association between sleep and physical functional impairment and disability requires establishing its temporality, an important criterion according to Bradford Hill to consider causation between two variables (13). All previous studies on this matter except three (6,7,10) used a cross-sectional design. One of the mentioned studies was a pilot study (6), therefore used a small sample size, and another was performed only with men (10). The study of Ensrud et al. (2012) (7), although using several types of sleep measures and a large sample, evaluated only frailty and mortality. None of the three studies explored many dimensions of functional condition and sleep characteristics (duration and quality) using the number of variables used in this study.

Also, sleep quantity and quality should be assessed, considering that prospective studies are not consistent if physical function outcomes are associated only with sleep duration, sleep quality, or both (6,7,10). Finally, studies on this topic should ascertain all domains of physical impairment and disability, from self-reported to objective performance and disability in activities of daily living, to characterize the full impact of sleep on the different stages of physical decline. Therefore, the objective of this study was to examine the prospective association of sleep duration and quality with a range of measures of impairment in physical function and disability in older adults. To explore this possibility, we used data from two independent prospective cohort studies conducted in two countries with different lifestyle characteristics: The Seniors-ENRICA (Study on Nutrition and Cardiovascular Risk Factors in Spain) and the ELSA (English Longitudinal Study of Aging) cohorts.

## MATERIALS AND METHODS

### Study design and participants

The Seniors-ENRICA cohort was established in 2008-2010 with community-dwelling individuals aged 60 years and older, and methods have been reported elsewhere (14). The analyses were conducted with 1,773 individuals, and, when evaluating each outcome, we excluded individuals who had the outcome at baseline. Study participants gave written informed consent. The study was approved for the Clinical Research Ethics Committee of the La Paz University Hospital in Madrid.

Established in 2002-2003, ELSA is a biennial longitudinal study representative of people aged 50 and older living in private households in England (15). For this study, we used information from 4,885 participants aged ≥60 years who participated in wave 4 (2008-2009) and were followed through wave 6 (2012-2013). For each outcome, we excluded from analyses participants who had the outcome at baseline. The National Research Ethics Service (MREC/01/2/91) provided the ethical approval for ELSA.

### Sleep duration and sleep quality

Sleep duration was categorized as short sleep (≤6 h), normal sleep (7-8 h) and long sleep (≥9 h) (16). Change in sleep duration was calculated by subtracting the number of hours/night slept at follow-up from those slept at baseline, and classified as no change, increase or decrease using 30 minutes as threshold. General quality of sleep was classified as ‘poor sleep’ and ‘good sleep’, and difficulty falling asleep and awakening during the night were also reported. ‘Need to sleep at daytime’ and ‘not feeling rested in the morning’ were recorded in Seniors-ENRICA study, and ‘wake up feeling tired or worn up’ in ELSA study. For the analyses, a sleep complaint was considered absent when rated as ‘rarely or never’, and was deemed to be present when it was reported to occur ‘sometimes’ or ‘almost always’ in Seniors-ENRICA study. In ELSA study, a sleep complaint was considered absent when its frequency was rated as ‘not during the last month’ or ‘less than once a week’, and was deemed to be present when it was reported to occur ‘once or twice a week’ or ‘three or more times a week’. Given that the simultaneous presence of many sleep complaints is common in older adults, and is associated with worse health (17), we calculated a ‘dyssomnia score’, whose range was from 0 to 4 (Seniors-ENRICA study) or 0-3 (ELSA study) of the above sleep complaints (12).

### Physical function impairment and disability

In the seniors-ENRICA study, individuals were defined as having impaired agility when they answered “a lot” to the following question from the Rosow and Breslau scale (18): “On an average day with your current health, would you be limited in bending and kneeling?”, whose categories of response were “yes, a lot”, “yes, a little” and “not at all”. In the ELSA study, the question used was “Do you experience any difficulty stooping, kneeling or crouching?”

**Table 1:** Characteristics of the participants in the Seniors-ENRICA study at baseline according to physical function impairment and disability.

	Self-reported impaired agility		Self-reported impaired mobility		5-point decrease in PCS		SPPB score ≤9 points		IADL disability		ADL disability	
	(n=1,293)		(n=1,475)		(n= 1,627)		(n=665)		(n=1,308)		(n= 1,249)	
	No	Yes	No	Yes	No	Yes	No	Yes	No	Yes	No	Yes
Participants, n	1099	194	1298	177	1135	492	436	229	1203	105	1120	129
Female, %	42.0	55.2	43.2	71.75	49.3	56.5	40.8	43.7	48.9	62.9	46.3	67.4
Age, y	70.0 (5.2)	72.7 (5.8)	70.5 (5.6)	73.4 (6.2)	71 (6.0)	71.7 (5.9)	68.7 (4.6)	71.2 (5.8)	70.3 (5.3)	75.6 (6.9)	70.5 (5.6)	73.1 (6.3)
Educational level, %												
Primary	41.5	55.1	44.9	62.2	47.3	53.2	39.5	48.0	47.6	68.6	48.1	55.8
Secondary	30.4	23.2	28.6	20.8	27.8	25.5	29.1	22.7	27.0	16.2	25.2	26.4
University	28.1	21.7	26.5	17.0	24.9	21.3	31.4	29.3	25.4	15.2	26.7	17.8
Current smokers, %	9.9	8.3	9.7	6.2	9.3	8.5	11.9	8.3	9.0	12.4	9.7	8.5
Alcohol intake ^*^, %												
None	28.1	46.3	31.7	48.7	33.9	40.7	29.4	36.7	33.2	49.5	33.1	42.6
Moderate	65.7	49.1	62.8	46.8	61.3	54.0	64.4	55.9	60.8	50.5	61.4	51.2
Heavy	6.2	4.6	5.5	4.5	4.8	5.3	6.2	7.4	6.0	0.0	5.5	6.2
Energy intake, Kcal/d	2083 (586)	1951 (423)	2073 (569)	1954 (407)	2052 (531)	2020 (594)	2120 (594)	2096 (722)	2070 (571)	1995 (466)	2064 (576)	2026 (416)
BMI, kg/m^2^	27.5 (3.7)	29.2 (4.3)	27.9 (3.8)	28.9 (4.4)	28.4 (4.2)	28.7 (4.3)	27.8 (3.9)	28.3 (3.8)	28.3 (4)	29.3 (5.5)	28.1 (4)	29.8 (4.7)
Caffeine consumption, mg/d	94 (137)	74 (128)	89 (1345)	86 (206)	91 (144)	82 (165)	96 (146)	112 (211)	92 (152)	85 (123)	91 (153)	99 (150)
Physical activity, MET-h/wk	25.3 (15.1)	21.4 (13.1)	24.5 (15.1)	19.2 (12.1)	23.4 (15.1)	21.0 (14.3)	26.4 (16.4)	24.7 (14.6)	23.6 (15.2)	16.5 (11.3)	23.3 (15.2)	19.0 (13.3)
Time spent watching TV, h/wk	18.4 (30.7)	19.8 (10.6)	19.1 (28.7)	19.5 (10.1)	20.4 (42.0)	19.9 (10.4)	19.6 (47.5)	18.2 (9.7)	19.9 (40.8)	22.4 (10.5)	20.1 (42.2)	20.1 (10.9)
Diagnosed diseases, %												
Cardiovascular disease ^†^	4.1	6.2	4.6	6.8	5.4	6.3	3.0	5.2	4.8	9.5	4.6	10.1
Osteomuscular disease ^‡^	34.6	61.9	39.8	65.5	45.9	53.5	35.6	55.5	45.1	61.0	44.9	58.9
Diabetes	16.8	21.7	18.1	23.2	18.0	22.4	14.7	21.8	17.8	24.8	18.0	24.8
Cancer	2.6	2.1	2.5	2.8	2.6	2.4	1.8	2.2	2.7	1.9	2.6	1.6
Depression	5.0	6.2	5.8	8.5	7.4	9.2	3.7	5.2	6.3	14.3	7.1	10.1
Sleep duration, h	7.0 (1.2)	6.8 (1.4)	7.0 (1.3)	6.8 (1.4)	6.9 (1.3)	6.9 (1.4)	67.0 (1.3)	6.9 (1.3)	6.9 (1.0)	6.9 (1.5)	6.9 (1.3)	6.6 (1.4)
Poor overall sleep quality, %	21.5	36.6	23.6	36.7	28.7	32.7	23.4	30.1	28.3	38.1	27.4	41.1
Dyssomnia score ≥1, %	71.5	78.4	72.3	89.3	74.3	82.5	68.1	78.6	75.1	85.7	75.1	85.3

PCS: physical component summary; SPPB: Short Physical Performance Battery; IADL: instrumental activities of daily living; ADL: basic activities of daily living For continuous variables, the mean (standard deviation) is reported.

* Moderate drinker: <40 g/d of alcohol in men and <24 g/d in women. Heavy drinker: ≥40 g/d of alcohol in men and ≥24 g/d in women.

† Ischemic heart disease, stroke, and heart failure.

‡ Osteo-arthritis, arthritis, and hip fracture.

In the Seniors-ENRICA study, mobility disability was defined as answering “a lot” to any of the following questions from the Rosow and Breslau scale (18): “On an average day with your current health, would you be limited in the following activities: 1) picking up or carrying a shopping bag?; 2) climbing one flight of stairs?; 3) walking several city blocks (a few hundred meters)?” In the ELSA study, it was defined as an affirmative answer to at least one of the following: 1) “Do you experience difficulty lifting or carrying weights over 10 pounds?” 2) “Do you experience difficulty climbing one flight of stairs without resting?”, or 3) “Do you experience any difficulty walking 1/4 mile unaided?” Physical function impairment was evaluated using the Spanish version of the physical component summary (PCS) of the 12-Item Short-Form Health questionnaire (SF-12, version 2) (19), and the Short Physical Performance Battery (SPPB) (1). The PCS consists in a physical health score with the following items: physical functioning, role - physical, bodily pain, general health. The questionnaire uses Likert scales to analyze the intensity or frequency of each response. Physical function impairment was deemed to exist when the score decreased at least 5 points from baseline to follow-up. The Short Physical Performance Battery (SPPB) (1) is based on direct, objective measures of walking speed, balance, and lower body strength. Each measure is scored from 0 to 4, and participants scoring ≤9 were classified as physically impaired (11). Disability in performing instrumental activities of daily living (IADLs) (20) and basic activities of daily living (ADLs) (21) were also measured in the Seniors-ENRICA study.

**Table 2:** Characteristics of the participants in the ELSA study at baseline according to physical function impairment.

	Self-reported impaired agility		Self-reported impaired mobility	
	(n=2,053)		(n=2,348)	
	No	Yes	No	Yes
Participants, n (weighted)	1542	511	1947	401
Female, %	46.8	57.0	45.2	62.6
Age, y	67.9 (6.9)	71.3 (7.9)	68.0 (6.7)	72.7 (8.6)
Educational level, %				
No qualifications	25.8	35.5	24.3	36.7
Intermediate or foreign	31.3	34.0	32.9	35.5
Higher education	42.9	30.5	42.8	27.9
Current smokers, %	8.1	8.1	7.3	5.3
Alcohol intake				
0-2 times/month	31.6	41.1	31.8	41.1
1-4 times/week	42.0	35.7	41.1	37.0
5-7 times/week	26.3	23.2	27.1	21.9
BMI, kg/m^2^	26.9 (4.0)	28.3 (5.0)	27.6 (4.3)	28.7 (4.9)
Physical activity, %				
hardly ever/never	27.1	15.2	27.1	11.9
1-3 times/ month	55.9	52.7	56.7	60.2
once/ week	14.3	28.1	14.3	24.3
> once/week	2.7	4.1	2.0	3.5
Time spent watching TV, h/wk	41.1 (33.5)	46.5 (36.9)	41.4 (32.2)	45.0 (36.7)
Diagnosed diseases, %				
Cardiovascular disease ^*^	0.7	1.4	0.8	0.9
Osteomuscular disease ^†^	15.2	28.8	19.7	37.4
Diabetes	7.5	12.9	7.3	12.4
Cancer	1.9	2.3	2.1	3.6
Depression	4.9	7.6	5.2	7.1
Sleep duration, h	7.0 (1.1)	6.9 (1.4)	7.0 (1.2)	6.8 (1.4)
Poor overall sleep quality, %	12.6	20.7	13.7	22.0
Dyssomnia score ≥1, %	67.5	77.2	69.3	77.9

For continuous variables, the mean (standard deviation) is reported.

* Ischemic heart disease, stroke, and heart failure.

† Osteo-arthritis, arthritis, and hip fracture.

### Other variables

At baseline in both cohorts, self-reported information was obtained on age, sex, educational status and tobacco consumption. Weight and height were measured in standardized conditions (22), and the body mass index (BMI) was calculated as weight in kg divided by square height in m and classified as <25, 25-29.9 and ≥30 kg/m^2^. Participants also reported the following physician-diagnosed diseases: cardiovascular disease (ischemic heart disease, stroke and heart failure), diabetes, cancer, depression requiring pharmacological treatment, and osteomuscular diseases (osteoarthritis, arthritis and hip fracture). Sedentary behavior was approximated by the time (hours/week) spent watching TV.

In the Seniors-ENRICA study, physical activity during leisure time (metabolic equivalent hours/week) was ascertained with the EPIC-cohort questionnaire, validated in Spain (23). Total energy intake (kcal/d), caffeine and alcohol intake were estimated from a validated diet history and standard food composition tables (24). Alcohol consumption was categorized as no drinker, moderate drinker and heavy drinker (≥40 g/d of alcohol in men and ≥24 g/d in women) (25). In the ELSA study, participants informed the weekly frequency of alcohol consumption. Cognitive function was assessed in a subsample of participants of the Seniors-ENRICA study with the Mini-Mental State Examination (MMSE) (26). Information on sleeping medication (yes or no) was also collected in the Seniors-ENRICA study.

**Table 3:** Odds ratios (95% confidence interval)^a^ for the association between change in sleep duration and physical function impairment during a 2.8 year follow-up in Seniors-ENRICA study and a 2 year follow-up in the ELSA study of older adults.

	No change^b^	Decrease ≥30 min	Increase ≥30 min
Self-reported impaired agility			
Seniors-ENRICA study, N/n cases	458/51	350/56	485/87
Age- and sex-adjusted	1.00	1.43 (0.94; 2.17)	1.67 (1.14; 2.44)
Multivariable model^c^	1.00	1.37 (0.85; 2.21)	1.23 (0.80; 1.89)
Multivariable model^d^	1.00	1.35 (0.84; 2.19)	1.21 (0.78; 1.87)
Multivariable model^e^	1.00	1.35 (0.83; 2.19)	1.24 (0.80; 1.92)
ELSA study, N/n cases	741/183	641/164	671/164
Age- and sex-adjusted	1.00	1.01 (0.80; 1.28)	0.91 (0.71; 1.16)
Multivariable model^f^	1.00	1.01 (0.78; 1.29)	0.84 (0.65; 1.10)
Multivariable model^g^	1.00	0.98 (0.76; 1.27)	0.83 (0.63; 1.08)
Multivariable model^h^	1.00	0.96 (0.74; 1.24)	0.82 (0.63; 1.07)
Self-reported impaired mobility			
Seniors-ENRICA study, N/n cases	511/46	410/59	554/72
Age- and sex-adjusted	1.00	1.53 (1.00; 2.34)	1.42 (0.95; 2.13)
Multivariable model^c^	1.00	1.57 (0.97; 2.54)	1.32 (0.83; 2.09)
Multivariable model^d^	1.00	1.57 (0.97; 2.55)	1.31 (0.83; 2.08)
Multivariable model^e^	1.00	1.50 (0.92; 2.45)	1.31 (0.82; 2.08)
ELSA study, N/n cases	845/127	747/141	756/133
Age- and sex-adjusted	1.00	1.33 (1.02; 1.72)	1.15 (0.88; 1.52)
Multivariable model^f^	1.00	1.27 (0.96; 1.67)	1.04 (0.77; 1.40)
Multivariable model^g^	1.00	1.26 (0.95; 1.66)	1.02 (0.76; 1.38)
Multivariable model^h^	1.00	1.22 (0.92; 1.62)	1.01 (0.75; 1.36)
5-point decrease in PCS^i^, N/n cases (Seniors-ENRICA study)	557/163	457/143	613/186
Age- and sex-adjusted	1.00	1.10 (0.83; 1.45)	1.05 (0.82; 1.37)
Multivariable model^c^	1.00	1.12 (0.82; 1.53)	0.98 (0.73; 1.31)
Multivariable model^d^	1.00	1.14 (0.83; 1.56)	0.97 (0.72; 1.30)
Multivariable model^e^	1.00	1.12 (0.81; 1.54)	0.97 (0.72; 1.30)
Impaired lower extremity function^j^, N/n cases (Seniors-ENRICA study)	237/75	184/66	244/88
Age- and sex-adjusted	1.00	1.11 (0.73; 1.69)	1.23 (0.83; 1.81)
Multivariable model^c^	1.00	1.18 (0.75; 1.87)	1.24 (0.81; 1.90)
Multivariable model^d^	1.00	1.17 (0.74; 1.85)	1.28 (0.83; 1.97)
Multivariable model^e^	1.00	1.14 (0.72; 1.81)	1.29 (0.84; 1.99)
IADL disability, N/n cases (Seniors-ENRICA study)	445/29	374/34	489/42
Age- and sex-adjusted	1.00	1.20 (0.70; 2.05)	1.13 (0.68; 1.88)
Multivariable model^c^	1.00	1.04 (0.58; 1.90)	1.08 (0.61; 1.89)
Multivariable model^d^	1.00	1.05 (0.58; 1.92)	1.10 (0.62; 1.94)
Multivariable model^e^	1.00	1.04 (0.57; 1.90)	1.11 (0.63; 1.96)
ADL disability, N/n cases (Seniors-ENRICA study)	424/38	353/36	472/55
Age- and sex-adjusted	1.00	1.01 (0.62; 1.65)	1.25 (0.80; 1.95)
Multivariable model^c^	1.00	1.13 (0.67; 1.90)	1.18 (0.73; 1.90)
Multivariable model^d^	1.00	1.14 (0.68; 1.91)	1.18 (0.73; 1.90)
Multivariable model^e^	1.00	1.13 (0.67; 1.91)	1.19 (0.74; 1.92)

PCS: physical component summary; SPPB: Short Physical Performance Battery; IADL: instrumental activities of daily living; ADL: basic activities of daily living

a Logistic regression models.

b Changes < 30 min.

c Adjusted for educational level (primary, secondary, university), BMI at baseline and change (continuous, kg/m^2^), change in tobacco consumption (never smoker, former smoker in 2012, smoker in 2012 who quit in 2015, and current smoker in 2015), baseline adherence to the Mediterranean diet (MEDAS score, tertiles), baseline intakes of energy (kcal/day, tertiles), caffeine (g/day, tertiles) and alcohol (none, moderate, heavy drinker), baseline and change in time spent watching TV(h/wk, tertiles), baseline diagnosed diseases (cardiovascular diseases, diabetes, cancer, depression and osteomuscular diseases) and baseline sleep duration (≤6, 7-8, ≥9 h).

d Additionally adjusted for baseline and change in physical activity (METs*hours/week, tertiles).

e Additionally adjusted for general sleep quality (good, poor) and for the dyssomnia score (0, 1, 2, 3-4).

f Adjusted for educational level (no qualifications, intermediate or foreign, higher education), BMI at baseline and change (continuous, kg/m^2^), change in tobacco consumption (never smoker, former smoker in 2008, smoker in 2008 who quit in 2010, and current smoker in 2010), baseline intake of alcohol (5-7 times/week, 1-4 times/week, 0-2 times/month), baseline and change in time spent watching TV(h/wk, tertiles), baseline diagnosed diseases (cardiovascular diseases, diabetes, cancer, depression and osteomuscular diseases) and baseline sleep duration (≤6, 7-8, ≥9 h).

g Additionally adjusted for baseline physical activity (>once/week, once/week, 1-3 times/ month, hardly ever/never).

h Additionally adjusted for general sleep quality (good, poor) and for the dyssomnia score (0, 1, 2, 3).

i Additionally adjusted for basal PCS

j SPPB score ≤9 points.

#### Statistical analysis

We used logistic regression models to examine the association between changes in sleep duration and incident outcomes, using no change in sleep as reference. Likewise, logistic regression was used to assess the association of general sleep quality, each individual sleep complaint, and the dyssomnia score at baseline with the risk of physical function impairment and disability at follow-up. We also investigated the linear dose-response associations by using the number of sleep complaints as a continuous variable in the models. Study results were summarized with odds ratios (OR) and their corresponding 95% confidence interval (CI).

We built four logistic models. The first one was adjusted for age and sex, and the second one was further adjusted for educational level, lifestyle variables and morbidity. Because physical activity may play a key role on the development of physical function impairment (27), a third model was additionally adjusted for this variable. Lastly, a fourth model was additionally adjusted for sleep variables: analyses of changes in sleep duration were adjusted for general sleep quality and for the dyssomnia score at baseline, and analyses of general sleep quality and dyssomnia score were adjusted for changes in sleep duration.

We formally tested interaction between change in sleep duration and sleep quality by using likelihood-ratio tests, which compared models with and without cross-product interaction terms, finding no interaction between these two variables. Differences between men and women in the study associations were also tested with likelihood-ratio tests. As no interactions between sex and sleep variables in relation to physical function impairment and disability were found, results are presented for women and men combined. Statistical significance was set at 2-tailed p<0.05. The statistical analyses were performed using Stata software (version 13.0; Stata Corp., College Station).

**Table 4:** Odds ratios (95% confidence interval)^a^ for the association between poor general sleep quality and the dyssomnia score and physical function impairment during a 2.8-year follow-up in Seniors-ENRICA study and a 2-year follow-up in the ELSA study of older adults.

	Poor general sleep quality	Dyssomnia score				
		0	1	2	3-4 ^b^	p for trend
Self-reported impaired agility						
Seniors-ENRICA study, N/n cases	307/71	355/42	449/64	338/51	151/37	
Age- and sex-adjusted	2.11 (1.50; 2.96)	1.00	1.16 (0.76; 1.77)	1.09 (0.70; 1.71)	1.97 (1.19; 3.28)	0.04
Multivariable model^c^	1.89 (1.28; 2.78)	1.00	0.96 (0.60; 1.54)	1.02 (0.62; 1.69)	1.76 (0.99; 3.11)	0.09
Multivariable model^d^	1.94 (1.31; 2.87)	1.00	0.95 (0.59; 1.53)	1.00 (0.61; 1.66)	1.74 (0.98; 3.08)	0.10
Multivariable model^e^	1.93 (1.30; 2.86)	1.00	0.94 (0.59; 1.52)	0.99 (0.60; 1.64)	1.69 (0.95; 3.01)	0.13
ELSA study, N/n cases	299/106	618/117	438/122	686/89	520/114	
Age- and sex-adjusted	1.85 (1.42; 2.41)	1.00	1.42 (1.10; 1.84)	1.60 (1.20; 2.14)	2.53 (1.75; 3.65)	<0.001
Multivariable model^f^	1.65 (1.25; 2.17)	1.00	1.35 (1.03; 1.76)	1.55 (1.14; 2.09)	2.12 (1.44; 3.11)	<0.001
Multivariable model^g^	1.61 (1.22; 2.12)	1.00	1.35 (1.03; 1.76)	1.51 (1.11; 2.04)	2.06 (1.40; 3.03)	<0.001
Multivariable model^h^	1.65 (1.24; 2.18)	1.00	1.34 (1.03; 1.75)	1.53 (1.13; 2.06)	2.10 (1.43; 3.00)	<0.001
Self-reported impaired mobility	371/65	379/19	499/45	408/74	189/39	
Seniors-ENRICA study, N/n cases						
Age- and sex-adjusted	1.70 (1.20; 2.40)	1.00	1.75 (0.99; 3.06)	3.24 (1.89; 5.55)	3.33 (1.83; 6.04)	<0.001
Multivariable model^c^	1.39 (0.94; 2.06)	1.00	1.37 (0.76; 2.48)	2.32 (1.31; 4.11)	2.29 (1.20; 4.36)	0.002
Multivariable model^d^	1.45 (0.98; 2.16)	1.00	1.34 (0.74; 2.42)	2.30 (1.30; 4.09)	2.31 (1.21; 4.41)	0.001
Multivariable model ^e^	1.46 (0.98; 2.17)	1.00	1.33 (0.73; 2.40)	2.24 (1.26; 3.99)	2.28 (1.19; 4.37)	0.002
ELSA study, N/n cases	354/88	805/200	192/73	917/142	226/57	
Age- and sex-adjusted	1.79 (1.35; 2.36)	1.00	1.31 (0.97; 1.75)	1.92 (1.40; 2.62)	2.11 (1.41; 3.14)	<0.001
Multivariable model^f^	1.60 (1.19; 2.15)	1.00	1.25 (0.93; 1.69)	1.82 (1.30; 2.53)	1.79 (1.18; 2.71)	<0.001
Multivariable model^g^	1.58 (1.18; 2.13)	1.00	1.25 (0.92; 1.69)	1.79 (1.28; 2.50)	1.76 (1.16; 2.67)	<0.001
Multivariable model^h^	1.59 (1.18; 2.15)	1.00	1.25 (0.92; 1.69)	1.78 (1.28; 2.49)	1.76 (1.16; 2.68)	<0.001
5-point decrease in PCS^i^, N/n cases (Seniors-ENRICA study)	487/161	378/86	522/168	460/149	267/89	
Age- and sex-adjusted	1.47 (1.15; 1.88)	1.00	1.67 (1.23; 2.28)	1.70 (1.23; 2.34)	2.17 (1.48; 3.17)	<0.001
Multivariable model^c^	1.29 (0.98; 1.69)	1.00	1.56 (1.12; 2.18)	1.55 (1.09; 2.20)	1.82 (1.19; 2.76)	0.008
Multivariable model^d^	1.38 (1.05; 1.81)	1.00	1.52 (1.09; 2.14)	1.53 (1.07; 2.19)	1.87 (1.22; 2.86)	0.006
Multivariable model^e^	1.39 (1.05; 1.83)	1.00	1.52 (1.09; 2.14)	1.54 (1.08; 2.20)	1.89 (1.23; 2.89)	0.005
Impaired lower extremity function^j^, N/n cases (Seniors-ENRICA study)	171/69	188/49	231/74	179/78	67/28	
Age- and sex-adjusted	1.46 (1.00; 2.12)	1.00	1.30 (0.84; 2.02)	2.00 (1.27; 3.16)	1.94 (1.06; 3.56)	0.002
Multivariable model^c^	1.28 (0.85; 1.92)	1.00	1.14 (0.72; 1.81)	1.90 (1.16; 3.12)	1.38 (0.71; 2.68)	0.04
Multivariable model^d^	1.26 (0.84; 1.89)	1.00	1.15 (0.72; 1.84)	1.91 (1.16; 3.14)	1.36 (0.70; 2.65)	0.04
Multivariable model^e^	1.25 (0.83; 1.88)	1.00	1.16 (0.73; 1.84)	1.91 (1.16; 3.14)	1.34 (0.68; 2.60)	0.05
IADL disability, N/n cases (Seniors-ENRICA study)	380/40	315/15	433/32	358/34	202/24	
Age- and sex-adjusted	1.72 (1.11; 2.68)	1.00	1.38 (0.72; 2.64)	1.59 (0.83; 3.05)	1.96 (0.97; 3.96)	0.05
Multivariable model^c^	1.59 (0.98; 2.58)	1.00	1.19 (0.60; 2.37)	1.39 (0.69; 2.78)	1.37 (0.63; 2.95)	0.37
Multivariable model^d^	1.57 (0.96; 2.54)	1.00	1.14 (0.57; 2.28)	1.30 (0.65; 2.64)	1.25 (0.57; 2.71)	0.04
Multivariable model^e^	1.59 (0.97; 2.59)	1.00	1.14 (0.57; 2.29)	1.30 (0.64; 2.64)	1.26 (0.58; 2.75)	0.51
ADL disability, N/n cases (Seniors-ENRICA study)	360/53	298/19	415/43	355/41	181/26	
Age- and sex-adjusted	1.71(1.16; 2.53)	1.00	1.55 (0.88; 2.74)	1.50 (0.84; 2.67)	1.74 (0.91; 3.30)	0.14
Multivariable model^c^	1.76 (1.16; 2.67)	1.00	1.42 (0.79; 2.55)	1.39 (0.76; 2.55)	1.49 (0.75; 2.95)	0.31
Multivariable model^d^	1.75 (1.15; 2.67)	1.00	1.39 (0.77; 2.51)	1.36 (0.74; 2.51)	1.44 (0.72; 2.87)	0.51
Multivariable model^e^	1.73 (1.14; 2.64)	1.00	1.38 (0.77; 2.49)	1.35 (0.73; 2.48)	1.40 (0.70; 2.80)	0.42

PCS: physical component summary; SPPB: Short Physical Performance Battery; IADL: instrumental activities of daily living; ADL: basic activities of daily living

a Logistic regression models.

b 3 sleep complaints For ELSA study.

c Adjusted for educational level (primary, secondary, university), BMI at baseline and change (continuous, kg/m^2^), change in tobacco consumption (never smoker, former smoker in 2012, smoker in 2012 who quit in 2015, and current smoker in 2015), baseline adherence to the Mediterranean diet (MEDAS score, tertiles), baseline intakes of energy (kcal/day, tertiles), caffeine (g/day, tertiles) and alcohol (none, moderate, heavy drinker), baseline and change in time spent watching TV(h/wk, tertiles) and baseline diagnosed diseases (cardiovascular diseases, diabetes, cancer, depression and osteomuscular diseases).

d Additionally adjusted for baseline and change in physical activity (METs*hours/week, tertiles).

e Additionally adjusted for change in sleep duration (no change, decrease, increase).

f Adjusted for educational level (no qualifications, intermediate or foreign, higher education), BMI at baseline and change (continuous, kg/m^2^), change in tobacco consumption (never smoker, former smoker in 2008, smoker in 2008 who quit in 2010, and current smoker in 2010), baseline intake of alcohol (5-7 times/week, 1-4 times/week, 0-2 times/month), baseline and change in time spent watching TV(h/wk, tertiles), baseline diagnosed diseases (cardiovascular diseases, diabetes, cancer, depression and osteomuscular diseases) and baseline sleep duration (≤6, 7-8, ≥9 h).

g Additionally adjusted for baseline physical activity (>once/week, once/week, 1-3 times/ month, hardly ever/never).

h Additionally adjusted for change in sleep duration (no change, decrease, increase).

i Additionally adjusted for basal PCS.

j SPPB score ≤9 points.

## RESULTS

During a mean follow-up of 2.8 years in the Seniors-ENRICA study and 2 years in the ELSA study, the incidence of limitations in agility in each cohort was 15.0% and 25.0%; and mobility disability was 12.0% and 17.0%, respectively. In the Seniors-ENRICA study, the incidence of impaired overall physical performance was 30.2%. Moreover, 34.0% of the individuals developed impaired lower extremity function at the end of follow-up, 8% developed IADL disability and 10.3% developed ADL disability.

Tables 1 and 2 show the sociodemographic, behavioral and clinical characteristics of the participants in the Seniors-ENRICA study and the ELSA study at baseline according to categories of physical function impairment or disability. Compared to those not impaired, those with limitation in any of the seven studied domains were older, mostly women, and with lower educational level. Also, they had higher BMI, and more frequently had cardiovascular and osteomuscular diseases, diabetes and depression. In the Seniors-ENRICA study, those participants also did less physical activity.

Mean (SD) sleep duration in the study participants was 6.9 (1.4) and 6.9 (1.3) h/night at baseline and 7.0 (1.4) and 6.9 (1.3) h/night at follow-up in Seniors-ENRICA study and in ELSA study, respectively. During the follow-up, 500 (28.2%) and 1031 (31.0%) persons decreased their sleep duration at least 30 minutes/night in Seniors-ENRICA study and in ELSA study, respectively. In addition, 676 (38.1%) and 1086 (33.7%) persons reported an increase of at least 30 minutes/night in Seniors-ENRICA study and in ELSA study, respectively. No association was seen among decrease or increase in the sleep duration and the studied outcomes (Table 3).

The associations between sleep quality and study outcomes are shown in Table 4. Overall, in Senior-ENRICA study, 29% of the participants reported poor general sleep quality, and 77% had at least one of the four sleep complaints considered, 44% at least two, and 17% three or four. In ELSA study, 20% of the participants reported poor general sleep quality, and 36% had at least one of the three sleep complaints, 25% at least two, and 14% all three. In both studies, poor general sleep quality was associated with higher risk of impaired agility [OR: 1.93 (95% CI: 1.30-2.86) in Seniors-ENRICA and 1.65 (1.24-2.18) in ELSA study] and mobility disability [1.46 (0.98-2.17) in Seniors-ENRICA and 1.59 (1.18-2.15) in ELSA study]. Poor general sleep quality was also associated with decreased PCS [1.39 (1.05-1.83)], IADL disability [1.59 (0.97-2.59)] and ADL disability [1.73 (1.14-2.64)].

In Seniors-ENRICA study, compared to those with no sleep complaints, participants with 2 and 3-4 complaints had greater risk of mobility disability [2.24 (1.26-3.99) and 2.28 (1.19-4.37), respectively; p-trend=0.002], decreased PCS [1.54 (1.08-2.20) and 1.89 (1.23-2.89), respectively; p-trend=0.005] and impaired lower extremity function [1.91 (1.16-3.14) and 1.34 (0.68-2.60), respectively, p-trend=0.05]. Additional adjustment for sleeping medication did not materially change the results (data not shown). The associations between the individual components of the dyssomnia score and the studied outcomes in Seniors-ENRICA study are shown in Table 5. Finally, as a sensitivity analyses, we replicated the regression models with further adjustment for cognitive impairment and the results did not materially change. In ELSA study, compared to those with no sleep complaints, participants with 2 and 3 complaints had greater risk of impaired agility [1.53 (1.13-2.06) and 2.10 (1.43-3.00), respectively; p-trend<0.001] and mobility disability [1.78 (1.28-2.49) and 1.76 (1.16-2.68), respectively; p-trend<0.001]. The associations between the individual components of the dyssomnia score and the studied outcomes in ELSA study are shown in Table 6.

**Table 5:** Odds ratios (95% confidence interval) ^*^ for the association between indicators of poor sleep quality and physical function impairment during a 2.8-year follow-up in Seniors-ENRICA study.

	Difficulty falling asleep	Awakening during the night	Need to sleep at daytime	Not feeling rested in the morning
Self-reported impaired agility, N/n cases	394/72	800/126	175/32	241/57
Age- and sex-adjusted	1.19 (0.85; 1.66)	1.07 (0.77; 1.48)	1.32 (0.86; 2.02)	1.86 (1.30; 2.67)
Multivariable model^†^	1.20 (0.82; 1.74)	1.02 (0.71; 1.48)	1.31 (0.81; 2.11)	1.75 (1.17; 2.62)
Multivariable model^‡^	1.19 (0.82; 1.74)	1.01 (0.70; 1.46)	1.29 (0.80; 2.08)	1.75 (1.17; 2.63)
Multivariable model^§^	1.17 (0.80; 1.71)	1.00 (0.69; 1.44)	1.25 (0.77; 2.03)	1.75 (1.16; 2.63)
Self-reported impaired mobility, N/n cases	483/87	933/136	222/44	282/52
Age- and sex-adjusted	1.59 (1.14; 2.22)	1.80 (1.24; 2.63)	2.04 (1.38; 3.02)	1.59 (1.10; 2.29)
Multivariable model^†^	1.30 (0.90; 1.89)	1.44 (0.96; 2.16)	1.75 (1.12; 2.71)	1.50 (1.00; 2.27)
Multivariable model^‡^	1.33 (0.91; 1.93)	1.42 (0.94; 2.14)	1.74 (1.12; 2.71)	1.53 (1.01; 2.30)
Multivariable model^§^	1.31 (0.90; 1.90)	1.40 (0.93; 2.10)	1.74 (1.11; 2.71)	1.54 (1.02; 2.33)
5-point decrease in PCS^||^, N/n cases	593/209	1070/325	257/86	383/120
Age- and sex-adjusted	1.5 (1.19; 1.90)	1.41 (1.11; 1.78)	1.31 (0.97; 1.76)	1.29 (0.98; 1.68)
Multivariable model^†^	1.37 (1.06; 1.77)	1.27 (0.98; 1.65)	1.24 (0.9; 1.71)	1.21 (0.90; 1.63)
Multivariable model^‡^	1.39 (1.07; 1.81)	1.26 (0.97; 1.63)	1.22 (0.88; 1.68)	1.27 (0.94; 1.71)
Multivariable model^§^	1.40 (1.07; 1.81)	1.26 (0.97; 1.64)	1.23 (0.88; 1.70)	1.28 (0.95; 1.72)
Impaired lower extremity function^¶^, N/n cases	202/88	390/143	88/37	121/52
Age- and sex-adjusted	1.71 (1.18; 2.47)	1.21 (0.87; 1.70)	1.35 (0.84; 2.16)	1.55 (1.03; 2.35)
Multivariable model^†^	1.64 (1.10; 2.45)	1.10 (0.77; 1.58)	1.23 (0.74; 2.04)	1.29 (0.82; 2.02)
Multivariable model^‡^	1.65 (1.10; 2.46)	1.11 (0.77; 1.59)	1.21 (0.73; 2.01)	1.26 (0.81; 1.98)
Multivariable model^§^	1.64 (1.09; 2.45)	1.10 (0.77; 1.58)	1.19 (0.71; 1.98)	1.27 (0.81; 1.99)
IADL disability, N/n cases	467/50	838/78	198/20	293/30
Age- and sex-adjusted	1.27 (0.83; 1.97)	1.49 (0.93; 2.39)	1.33 (0.78; 2.26)	1.31 (0.82; 2.09)
Multivariable model^†^	1.09 (0.69; 1.73)	1.38 (0.84; 2.30)	1.16 (0.65; 2.07)	1.06 (0.64; 1.76)
Multivariable model^‡^	1.07 (0.67; 1.71)	1.31 (0.79; 2.18)	1.10 (0.61; 1.97)	1.02 (0.61; 1.70)
Multivariable model^§^	1.07 (0.67; 1.71)	1.31 (0.79; 2.18)	1.11 (0.62; 1.99)	1.03 (0.61; 1.71)
ADL disability, N/n cases	436/56	798/96	196/18	278/41
Age- and sex-adjusted	1.15 (0.78; 1.70)	1.52 (1.00; 2.31)	0.82 (0.48; 1.40)	1.47 (0.98; 2.22)
Multivariable model^†^	1.14 (0.76; 1.72)	1.46 (0.94; 2.26)	0.67 (0.38; 1.18)	1.43 (0.92; 2.20)
Multivariable model^‡^	1.07 (0.67; 1.71)	1.44 (0.93; 2.23)	0.65 (0.37; 1.14)	1.40 (0.91; 2.17)
Multivariable model^§^	1.12 (0.74; 1.69)	1.43 (0.92; 2.21)	0.64 (0.36; 1.14)	1.39 (0.89; 2.15)

* Logistic regression models.

† Adjusted for educational level (primary, secondary, university), BMI at baseline and change (continuous, kg/m^2^), change in tobacco consumption (never smoker, former smoker in 2012, smoker in 2012 who quit in 2015, and current smoker in 2015), baseline adherence to the Mediterranean diet (MEDAS score, tertiles), baseline intakes of energy (kcal/day, tertiles), caffeine (g/day, tertiles) and alcohol (none, moderate, heavy drinker), baseline and change in time spent watching TV(h/wk, tertiles) and baseline diagnosed diseases (cardiovascular diseases, diabetes, cancer, depression and osteomuscular diseases).

‡ Additionally adjusted for baseline and change in physical activity (METs*hours/week, tertiles).

§ Additionally adjusted for change in sleep duration (no change, decrease, increase).

|| Additionally adjusted for basal PCS.

¶ SPPB score ≤9 points.

**Table 6:** Odds ratios (95% confidence interval)^*^ for the association between indicators of poor sleep quality and physical function impairment during a 2 year follow-up in the ELSA study of older adults.

	Poor sleep quality	Difficulty falling asleep	Awakening during the night	Waking up tired	Restless sleep
Self-reported impaired agility, N/n cases	299/106	453/1389	1308/364	496/161	539/174
Age- and sex-adjusted	1.85 (1.42;2.41)	1.37 (1.08;1.74)	1.52 (1.23;1.89)	1.64 (1.31;2.05)	1.64 (1.31;2.04)
Multivariable model ^†^	1.65 (1.25;2.17)	1.29 (1.00;1.65)	1.47 (1.17;1.84)	1.49 (1.18;1.89)	1.49 (1.18;1.88)
Multivariable model ^‡^	1.61 (1.22;2.12)	1.27 (0.99;1.63)	1.46 (1.16;1.83)	1.44 (1.13;1.83)	1.47 (1.16;1.85)
Multivariable model ^§^	1.65 (1.24;2.18)	1.29 (1.00;1.66)	1.47 (1.17;1.85)	1.46 (1.14;1.85)	1.49 (1.18;1.89)
Self-reported impaired mobility, N/n cases	354/88	530/117	1537/291	567/1354	643/145
Age- and sex-adjusted	1.79 (1.35;2.36)	1.30 (1.01;1.68)	1.49 (1.17;1.90)	1.82 (1.43;2.31)	1.57 (1.24;1.99)
Multivariable model ^†^	1.60 (1.19;2.15)	1.23 (0.94;1.61)	1.39 (1.08;1.80)	1.67 (1.30;2.16)	1.46 (1.14;1.88)
Multivariable model ^‡^	1.58 (1.18;2.13)	1.23 (0.94;1.61)	1.37 (1.06;1.77)	1.64 (1.27;2.13)	1.44 (1.12;1.84)
Multivariable model ^§^	1.59 (1.18;2.15)	1.23 (0.94;1.61)	1.37 (1.06;1.77)	1.64 (1.27;2.13)	1.44 (1.12;1.86)

* Logistic regression models.

† Adjusted for educational level (no qualifications, intermediate or foreign, higher education), BMI at baseline and change (continuous, kg/m^2^), change in tobacco consumption (never smoker, former smoker in 2008, smoker in 2008 who quit in 2010, and current smoker in 2010), baseline intake of alcohol (5-7 times/week, 1-4 times/week, 0-2 times/month), baseline and change in time spent watching TV(h/wk, tertiles), baseline diagnosed diseases (cardiovascular diseases, diabetes, cancer, depression and osteomuscular diseases) and baseline sleep duration (≤6, 7-8, ≥9 h).

‡ Additionally, adjusted for baseline physical activity (>once/week, once/week, 1-3 times/ month, hardly ever/never).

§ Additionally adjusted for change in sleep duration (no change, decrease, increase).

## DISCUSSION

In this prospective study of community-dwelling older adults from two European countries, general poor sleep quality and several sleep complaints were associated with higher risk of physical function impairment and disability. Regarding the prospective relation between sleep duration and quality and function outcomes, other studies diverge on the associations found. For instance, the study of Ensrud et al. (2012) (10) evaluated several sleep parameters, and found association between sleep quality variables and frailty and mortality at follow-up. Such associations did not occur for sleep duration. On the other hand, Lorenz et al. (2014) (6) evaluated only sleep duration and associated it with preclinical disability and mobility difficulty. The study of Stenholm et al. (2011) (7) also found relation between long sleep duration and long time spent in bed and decline in physical performance measured by the SPPB and subjective mobility disability.

The effect of sleep on health in the older population has been largely studied (28,29). However, the effect of changes in sleep duration over time is less known. Previous studies have found an association between changes in sleep (both increased and decreased) and higher risk of diabetes (30), lower cognitive function (31) and higher risk in premature mortality (32). Our results showed some tendency for both reduced and increased sleep to be associated with limited agility and mobility disability, but statistical significance was not reached possibly because of insufficient statistical power. Future research should assess further this hypothesis.

Individual sleep complaints had already shown a detrimental impact on physical function. For instance, poor general sleep quality was associated with impaired mobility (9) and frailty (10), and insomnia symptoms were associated with impaired mobility (6). Daytime complaints were linked to higher risk of social outcome, general productivity, vigilance, activity level, and global assessment of functional status of the Functional Outcomes of Sleepiness Questionnaire (FOSQ) (8), and frailty (11). When combining the sleep complaints, we found evidence of a cumulative effect on the risk of impaired agility, mobility disability, decreased PCS and impaired lower extremity function. Nighttime and daytime sleep symptoms are interconnected, mainly because sleep disturbances may cause daytime sleepiness (28,29). Nevertheless, nighttime symptoms are sometimes unrecognized or even considered normal in older adults, and some sleep disorders do not always cause full arousal (33). Our results suggest that isolated sleep complaints may not be able to characterize sleep quality and its impact on physical function. On the other hand, the single question on ‘self-reported poor general sleep quality’ was also a predictor of physical function impairment and disability, although the association was weaker than that observed for the dysomnia score. Corroborating, similar single questions about sleep quality were already associated with mobility disability (9) and loss of muscle mass (34).

Some mechanisms might explain the association between sleep and physical function impairment and disability. For example, poor sleep quality affect hypothalamus-pituitary-adrenal axis activity (35), resulting in hormone imbalances, such reduced growth hormone (36) and testosterone levels (37), and increased cortisol (38), favoring proteolysis and modifying body composition toward skeletal muscle loss (34). Furthermore, both sleep duration and quality play an important role on inflammation (39,40), and an elevated level of inflammation has been associated with poorer physical function in older adults (41).

Our study has several strengths, including the use of two distinct prospective cohorts from countries with different lifestyles, and that the results were robust after adjustment for many potential confounders, including physical activity during leisure time. Although we have used two cohorts, the totality of function variables was evaluated only in Seniors-ENRICA study. Limitations include the use of self-reported sleep duration and quality, which may be affected by recall and social desirability biases. However, the use of self-reported measures of sleep is habitual in large prospective studies due to its low-costs and simplicity. In addition, although objective measures of sleep duration are more accurate (42), information related to personal perception of sleep can only be achieved with self-reported data (43). Another limitation was the use of self-reported information for mobility disability, agility and the PCS; however, we combined it with a measure of lower extremity performance, in order to achieve a more complete assessment of physical function impairment. The relatively low incidence of the outcomes during the follow-up period may have limited the statistical power to detect the associations between sleep and some of the outcomes. Finally, as in most observational studies, certain residual confounding cannot be ruled out, despite adjustment for many variables.

### Conclusions

In conclusion, we found that accumulation of sleep complains in older individuals independently predict physical function impairment and disability. Screening sleep characteristics among the older people may be important to identify persons at risk of functional impairment.
